# Effect of hourly concentration of particulate matter on peak expiratory flow in hospitalized children: A panel study

**DOI:** 10.1186/1476-069X-10-15

**Published:** 2011-03-10

**Authors:** Shin Yamazaki, Masayuki Shima, Michiko Ando, Hiroshi Nitta, Hiroko Watanabe, Toshiyuki Nishimuta

**Affiliations:** 1 Department of Epidemiology and Healthcare Research, Kyoto University School of Public Health, Yoshidakonoe-cho, Sakyo-ku, Kyoto, Japan; 2 Department of Public Health, Hyogo College of Medicine, 1-1 Mukogawa-cho, Nishinomiya, Japan; 3 Department of Respirology, Graduate School of Medicine, Chiba University, 1-8-1 Inohana, Chuo-ku, Chiba, Japan; 4 Environmental Health Science Division, National Institute for Environmental Studies, 16-2 Onogawa, Tsukuba, Japan; 5 Department of Pediatrics, Shimoshizu National Hospital, 934-5 Shikawatashi, Yotsukaido, Japan

## Abstract

**Background:**

Little information is available on the possible association between hourly short-term air pollution and peak expiratory flow (PEF) in asthmatic children.

**Methods:**

PEF was measured twice daily, from October through December, 2000, in 17 children aged 8 to 15 years hospitalized with severe asthma. A total of 1198 PEF measurements were made at 7 a.m. and 1175 at 7 p.m. Measurements were conducted immediately prior to medication under the guidance of trained nurses. PEF changes were estimated in 10-μg/m^3 ^increments of particulate matter with a 50% cut-off aerodynamic diameter of ≤2.5 μm (PM_2.5_), with adjustment for sex, age, height, and temperature. Lagged-hour exposures of up to 24 hours were examined.

**Results:**

Increased 24-hour mean concentration of PM_2.5 _was associated with a decrease in both morning and evening PEF (-3.0 l/minute; 95%CI: -4.6, -1.4 and -4.4 l/minute; 95%CI: -7.1, -1.7, respectively). In addition, hourly concentrations of PM_2.5 _and PEF showed a significant association between some lags of PM_2.5 _and PEF. Effect size was almost -3 l/minute in both morning and evening PEF for an hourly PM_2.5 _concentration of 10 μg/m^3 ^in several lags. Even after adjustment for other air pollutants, some of the significant associations with PEF remained.

**Conclusion:**

Among hospitalized children with severe asthma, increased hourly concentration of PM_2.5 _was associated with a decrease in PEF.

## Background

Numerous studies across various environmental conditions have indicated the acute impact of ambient air pollution on human health [1]. In particular, elevated concentrations of particulate matter (PM) are associated with an increased incidence of respiratory symptoms and decreased lung function [1,2]. Children are considered to be more sensitive to air pollution than adults [3,4]. Environmental causes of asthma are usually related to climatic conditions that promote appreciable concentrations of atmospheric pollutants and antigens, and while any exposed individual in the general population may develop respiratory symptoms, the effect appears to more severe in those with pre-existing asthma or other respiratory diseases. Evidencing this, sensitivity to ambient PM differs between asthmatic and non-asthmatic children. Further, decrement of peak expiratory flow (PEF) in asthmatic children is associated with increased daily concentrations of PM [1]. In contrast, findings for the association between daily concentrations of PM and PEF in non-asthmatic children have been inconsistent, with few studies reporting results similar to those in asthmatic children [1].

Although asthmatic children appear more sensitive to PM than non-asthmatic children, the effect of transiently high concentrations of PM on PEF in these children remains unclear. Transiently high concentrations have been observed from hourly data, but not from daily data [5]. To identify possible associations between hourly short-term air pollution and PEF, we analyzed hourly air pollution and PEF data noted in the medical records of children hospitalized with asthma.

## Methods

### Subjects

The subjects of this panel study were 17 children aged 8 to 15 years who had been physician-diagnosed with severe asthma and were hospitalized at Shimoshizu National Hospital, Yotsukaido City, Japan. Yotsukaido City is located east of the greater Tokyo metropolitan area, within 40 kilometers of central Tokyo. This national hospital was established as a sanatorium in 1897, and is presently used primarily to provide long-term medical treatment. No major roads or factories are present in the vicinity of the hospital. Its large premises include a school for sick children adjacent to the hospital. Because the children had poorly controlled asthma with frequent exacerbations, they were under long-term hospitalization for maintenance of asthma medication and therefore attended the school. They are permitted to go outside when their condition is stable. All of the subjects had an atopic disposition and received asthma medication, including inhaled corticosteroids. In October 2000, informed written consent was obtained from all the subjects and their parents. The study was approved by the Medical Ethics Committee of Shimoshizu National Hospital.

### Outcome measurement

PEF was evaluated twice daily in all children using an electronic spirometer (AS-300; Minato Medical Inc., Tokyo, Japan). Measurements were conducted at 7 a.m. and 7 p.m., immediately prior to medication, under the guidance of trained nurses. PEF data were collected from October 1, 2000 through December 24, 2000. PEF was monitored to assess the efficacy of treatment for asthma. Because the children did not usually exhale from full inspiration to the maximal expiratory position, parameters such as forced expiratory volume in one second or forced vital capacity were not regularly recorded.

### Exposure assessment

Hourly concentrations of PM with a 50% cut-off aerodynamic diameter of ≤2.5 μm (PM_2.5_) were measured using an R&P TEOM-1400 (Rupprecht & Patashnick Co. Inc., Albany, NY) located at a monitoring station next to the hospital, at residential area. Data on hourly concentrations of nitrogen dioxide (NO_2_) and photochemical oxidants (Ox) measured at the monitoring station were obtained from the Chiba Prefectural Government. Local temperature data were obtained from the Japan Meteorological Agency. Concentrations of NO_2 _were measured by colorimetry using the Saltzman reagent method, while those of Ox were measured by absorption spectrophotometry using a neutral potassium iodide solution method according to guidelines of the Japanese Ministry of Environment. It is recognized that concentrations of Ox are nearly equivalent to those of ozone.

### Statistical methods

The association between hourly concentration of air pollutants and PEF was analyzed used Generalized Estimating Equations (GEE) [6]. We estimated the change in PEF by 10-μg/m^3 ^increments in PM_2.5 _with adjustment for sex, age, height at baseline survey, temperature at the time PEF was measured, day of the week and temporal trends (single-pollutant model). This basic model included sex, age, height and confounders selected on the basis of previous findings. We also estimated the change in PEF by 10-μg/m^3 ^increments in PM_2.5 _adjusted for hourly concentration of Ox, which was measured at the same time as PM_2.5_, and the variables described above (2-pollutant model), and adjusted for hourly concentrations of NO_2 _and Ox measured at the same time as PM_2.5_, as well as the variables above (3-pollutant model). Lagged-hour exposures of up to 24 hours were examined. For example, lag 12 for the PEF measured at 7 a.m. referred to the concentration of air pollutants during the period from 6 to 7 p.m. on the previous day, and lag 0 referred to the period from 6 to 7 a.m. on the same day. We also estimated previous 24-hour mean concentrations of air pollutants and PEF. Moreover, we also examined other air pollutants such as NO_2 _and Ox and PEF using a single-pollutant model and a 3-pollutant model.

Associations were estimated by GEE using the GENMOD procedure of SAS release 9.1 (SAS Institute, Inc., Cary, NC, USA). All tests were two-tailed, and alpha was set at 0.05. Changes in PEF and their 95% confidence intervals (CIs) were estimated.

## Results

**Figure 1:**
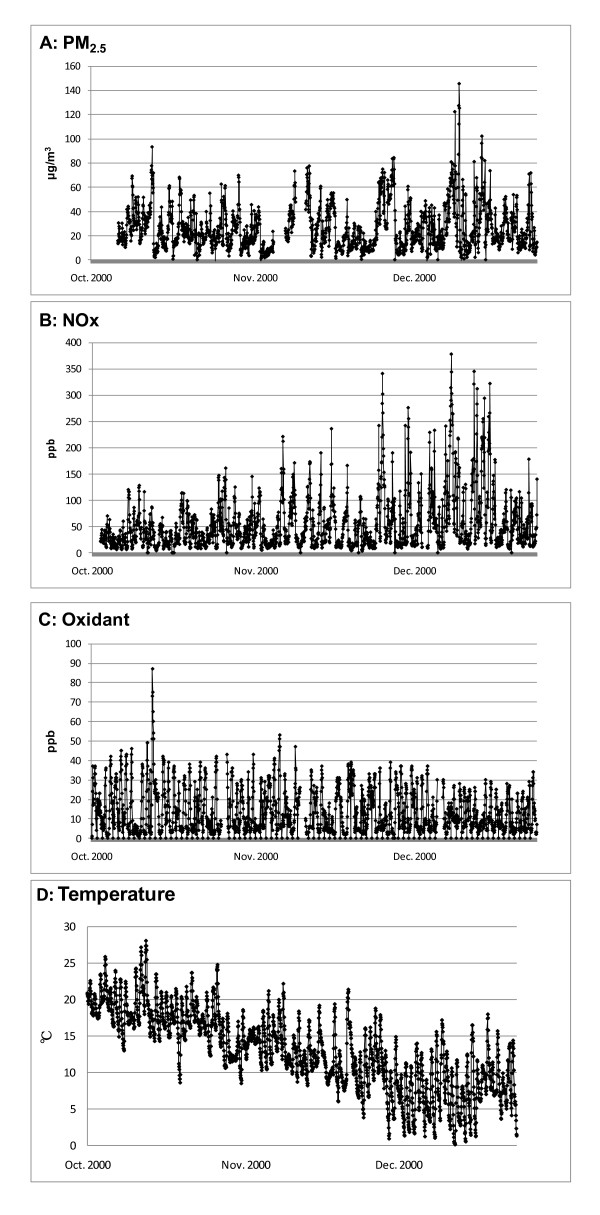
**Hourly concentration of air pollutants and hourly temperature from October 1, 2000 through December 24, 2000**. A: PM_2.5 _B: NOx C: Ox D: Temperature

Among the 17 asthmatic children, 1198 PEF measurements were conducted at 7 a.m. and 1175 at 7 p.m., giving an average of 70 morning and 69 evening measurements per child (Table 1). Table 1 also shows mean, minimum, and maximum PEF of each subject. Hourly mean concentrations of air pollutants at 7 a.m., 1 p.m., 7 p.m., and 1 a.m. are shown in Table 2. Correlations among these air pollutants are shown in Table 3. A longitudinal chart of hourly concentrations of PM_2.5 _(A), NOx (B), and Ox (C), and hourly temperature and relative humidity (D) is shown in Figure 1.

**Table 1:** Age, height, weight, mean peak expiratory flow (PEF), and number of PEF measurements for each subject

Sex	Age	Height	Weight	Number of PEF measurements		PEF		
				
				**7 a.m**.	**7 p.m**.	mean	max	min
	**years**	**cm**	**kg**	**n**	**n**	**l/minute**	**l/minute**	**l/minute**

Boy	8	119.0	20.3	64	67	217.6	329	105

Boy	9	133.2	33.3	58	63	250.6	326	138

Boy	9	136.7	32.3	67	70	260.9	436	142

Boy	10	137.0	33.3	72	72	305.9	434	219

Boy	10	138.3	28.6	68	65	198.2	358	130

Boy	10	130.8	25.7	70	67	301.9	427	126

Boy	11	134.5	29.3	72	71	232.5	349	106

Boy	11	145.3	35.9	77	74	288.0	415	123

Boy	12	163.0	53.7	65	66	473.1	669	386

Boy	12	134.5	30.2	66	68	296.1	382	160

Boy	13	160.4	46.3	77	70	378.7	450	281

Boy	14	164.0	56.9	77	74	505.3	692	193

Boy	15	170.5	51.7	81	75	399.6	530	181

Boy	15	163.2	60.0	68	61	459.6	561	301

Girl	10	139.1	31.4	73	72	294.2	397	188

Girl	12	152.5	51.5	70	71	351.4	474	214

Girl	12	147.2	41.8	73	69	241.9	319	145

Mean	11	145.2	39.0	71	69	320.9		

**Table 2:** Hourly mean concentration of air pollutants

		Morning		Noon		Evening		Night	
		
		(6 a.m. through 7 a.m.)		(12 p.m. through 1 p.m.)		(6 p.m. through 7 p.m.)		(12 a.m. through 1 a.m.)	
		
		Mean	(SD)	Mean	(SD)	Mean	(SD)	Mean	(SD)
PM_2.5_	(μg/m^3^)	24.0	(17.6)	26.9	(21.4)	30.0	(22.0)	25.8	(17.6)

Ox	(ppb)	8.3	(6.8)	23.3	(12.3)	13.1	(9.0)	9.1	(7.6)

NO_2_	(ppb)	24.0	(9.4)	22.2	(16.0)	32.6	(12.8)	28.3	(12.1)

Temperature	(°C)	10.1	(5.6)	15.9	(5.0)	14.2	(4.6)	11.6	(5.1)

PM_2.5_: Particulate matter with a 50% cut-off of aerodynamic diameter ≤2.5 μm

NO_2_: Nitrogen dioxide

Ox: Photochemical oxidants

**Table 3:** Correlation among air pollutants

	Correlation coefficient			
	
	Morning	Noon	Evening	Night
	
	(6 a.m. through 7 a.m.)	(12 p.m. through 1 p.m.)	(6 p.m. through 7 p.m.)	(12 a.m. through 1 a.m.)
PM_2.5 _- Ox	-0.44	-0.24	-0.27	-0.40

NO_2 _- PM_2.5_	0.54	0.78	0.62	0.56

Ox - NO_2_	-0.74	-0.53	-0.68	-0.72

PM_2.5_: Particulate matter with a 50% cut-off of aerodynamic diameter ≤2.5 μm

NO_2_: Nitrogen dioxide

Ox: Photochemical oxidants

### Association between hourly concentration of PM_2.5 _and PEF in the morning

**Figure 2:**
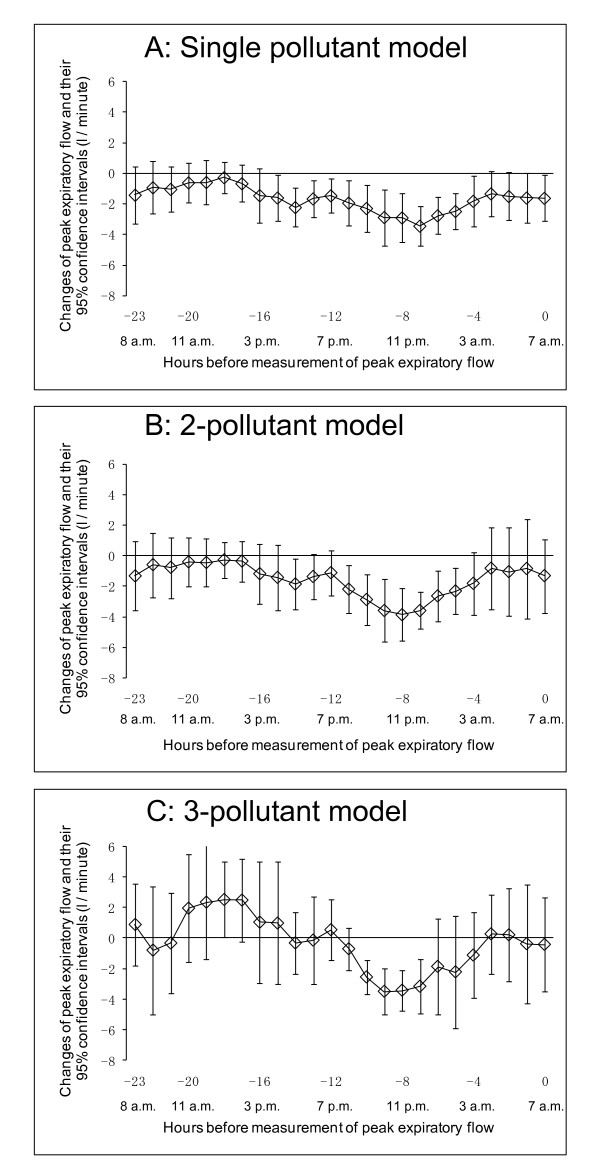
**Association between PEF measured in the morning at 7 a.m. and hourly concentration of PM_2.5_**. Lagged-hour exposures of up to 24 hours were examined. The mean differences and 95% confidence intervals in PEF per 10 μg/m^3 ^increases in PM_2.5 _were estimated. A: Single-pollutant model adjusted for age, sex, height, day of the week, temporal trends, and temperature. B: 2-pollutant model adjusted for age, sex, height, day of the week, temporal trends, photochemical oxidants, and temperature. C: 3-pollutant model adjusted for age, sex, height, day of the week, temporal trends, nitrogen dioxide, photochemical oxidants, and temperature.

Figure 2A shows the association between hourly concentration of PM_2.5 _and PEF at 7 a.m. using the single-pollutant model. A decline in PEF at 7 a.m. was associated with the hourly concentration of PM_2.5 _during the period from lag 15-hour to lag 4-hour, i.e., the period from 3 p.m. of the previous day to 3 a.m. of the same day in which PEF was measured. The largest effect size was -3.14 liters/minute (l/minute) (95% CI: -4.09, -2.20) for an hourly PM_2.5 _concentration of 10 μg/m^3 ^between 11 p.m. and 12 p.m. on the previous night (lag 7-hour). Some of the significant associations remained even after adjustment for other air pollutants using the multi-pollutant model (Figure 2B and Figure 2C).

### Association between hourly concentration of PM_2.5 _and PEF in the evening

**Figure 3:**
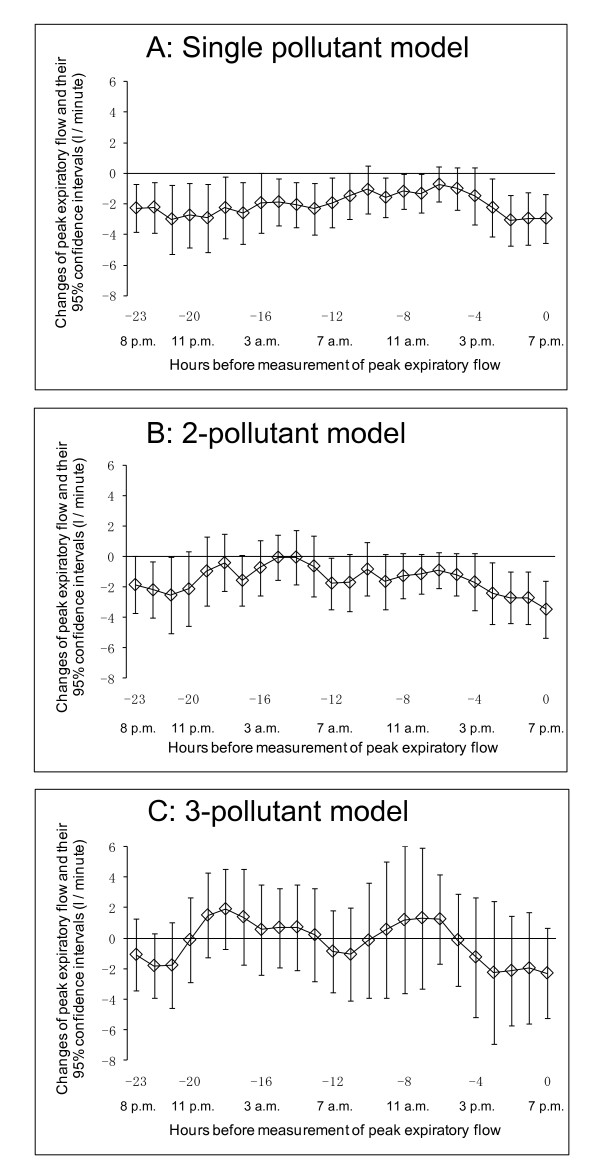
**Association between PEF measured in the evening at 7 p.m. and hourly concentration of PM_2.5_**. Lagged-hour exposures of up to 24 hours were examined. Mean differences and 95% confidence intervals in PEF for 10 μg/m^3 ^increases in PM_2.5 _were estimated. A: Single-pollutant model adjusted for age, sex, height, day of the week, temporal trends, and temperature. B: 2-pollutant model adjusted for age, sex, height, day of the week, temporal trends, photochemical oxidants, and temperature. C: 3-pollutant model adjusted for age, sex, height, day of the week, temporal trends, nitrogen dioxide, photochemical oxidants, and temperature.

Figure 3A shows the association between hourly concentration of PM_2.5 _and PEF at 7 p.m. using the single-pollutant model. A decline in PEF at 7 p.m. was associated with the hourly concentration of PM_2.5 _during the period from lag 3-hour to lag 0-hour, i.e., the period from 3 p.m. to 7 p.m. of the same day on which PEF was measured. The largest effect size was -3.06 l/minute (95% CI: -4.72, -1.41) for an hourly PM_2.5 _concentration of 10 μg/m^3 ^between 4 p.m. and 5 p.m. (lag 2-hour). This association was also seen during the period from lag 23-hour to lag 12-hour, i.e., the period from 7 p.m. of the previous day to 7 a.m. of the same day in which PEF were measured. Some of these significant associations remained on use of the 2-pollutant model (Figure 3B).

### Association between 24-hour concentration of PM_2.5 _and PEF

When we estimated the association between 24-hour concentration of PM_2.5 _and PEF, we observed a significant association in the single-pollutant model in both the morning and evening (-2.96 l/minute (95% CI: -4.55, -1.37) and -4.42 l/minute (95% CI: -7.11, -1.73) for a 24-hour mean PM_2.5 _concentration of 10 μg/m^3^, respectively) (Table 4). These associations were also seen with the 2-pollutant and 3-pollutant models, but without statistical significance except for the 2-pollutant model.

**Table 4:** Change in peak expiratory flow (PEF) for a 24-hour concentration of PM_2.5 _of 10 μ/m^3^

	Morning		Evening	
	
	(PEF at 7 a.m.)		(PEF at 7 p.m.)	
	
	Changes (l/minute)	(95%CI)	Changes (l/minute)	(95%CI)
Single pollutant model*	-2.96	(-4.55, -1.37)	-4.42	(-7.11, -1.73)

2-pollutant model†	-2.52	(-4.60, -0.44)	-3.51	(-6.37, -0.65)

3-pollutant model‡	-1.59	(-3.61, 0.43)	-2.36	(-7.59, 2.86)

PM_2.5_: Particulate matter with a 50% cut-off of aerodynamic diameter ≤2.5 μm

*Adjusted for sex, age, height, day of the week, temporal trends and temperature.

†Adjusted for photochemical oxidant, sex, age, height, day of the week, temporal trends and temperature.

‡Adjusted for nitrogen dioxide, photochemical oxidant, sex, age, height, day of the week, temporal trends and temperature.

### Association between hour concentrations of the other pollutants and PEF

**Figure 4:**
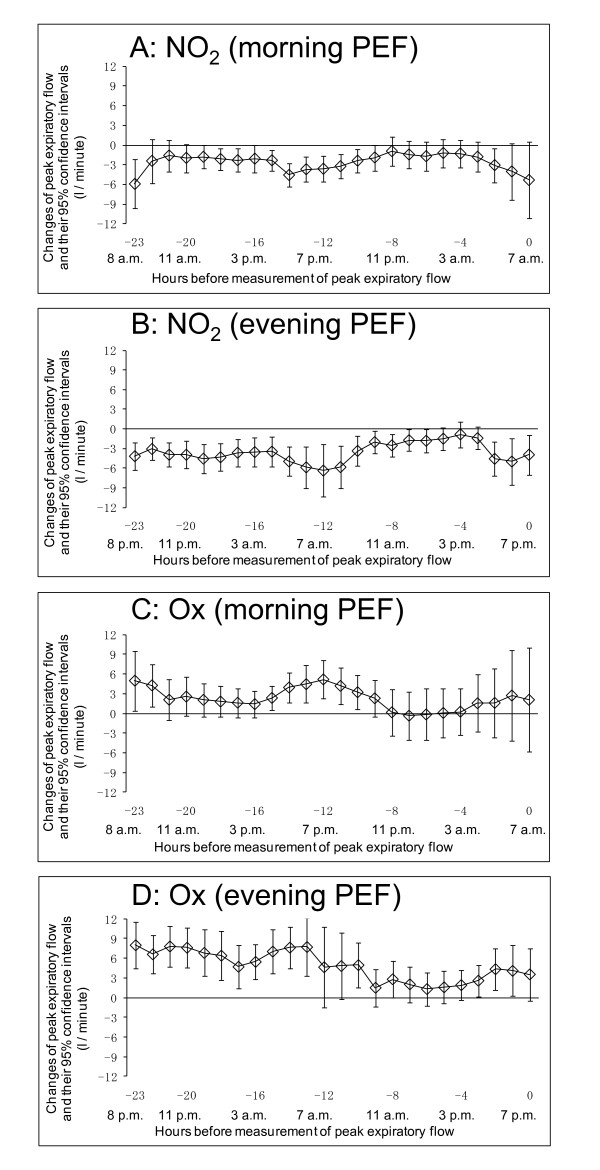
**Association between PEF and hourly concentration of other pollutants (NO_2 _and Ox) using single-pollutant model**. Lagged-hour exposures of up to 24 hours were examined. Mean differences and 95% confidence intervals in PEF for 10 ppb increases in NO_2_/Ox were estimated using single pollutant model adjusted for age, sex, height, day of the week, temporal trends, and temperature. A: Association between PEF measured in the morning at 7 a.m. and hourly concentration of NO_2_. B: Association between PEF measured in the evening at 7 p.m. and hourly concentration of NO_2_. C: Association between PEF measured in the morning at 7 a.m. and hourly concentration of Ox. D: Association between PEF measured in the evening at 7 p.m. and hourly concentration of Ox

**Figure 5:**
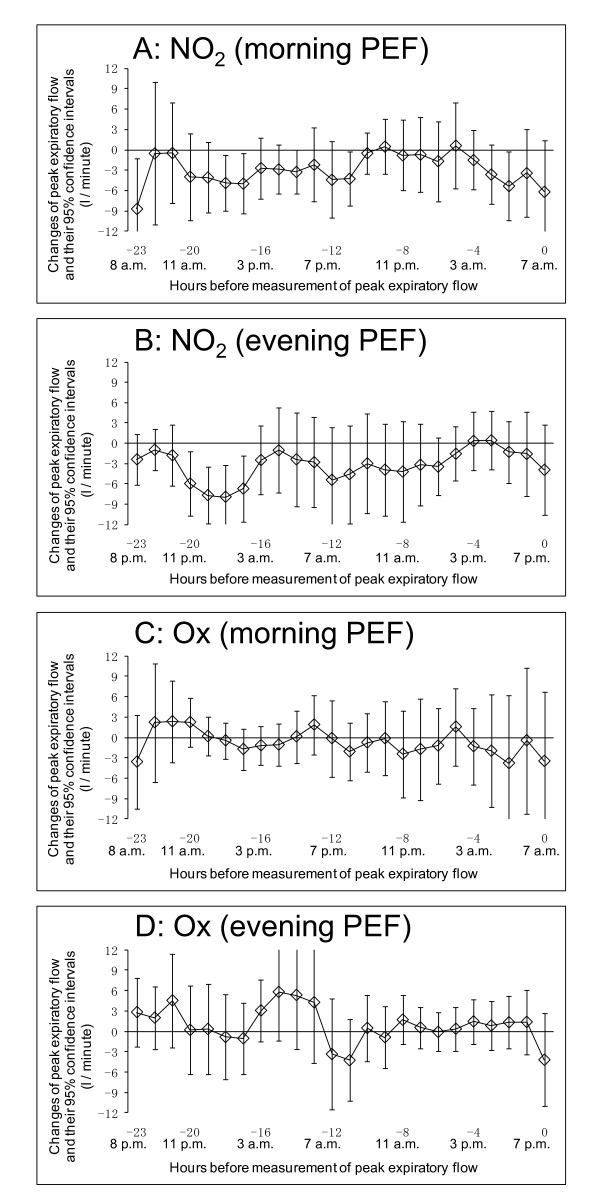
**Association between PEF and hourly concentration of other pollutants (NO_2 _and Ox) using 3-pollutant model**. Lagged-hour exposures of up to 24 hours were examined. Mean differences and 95% confidence intervals in PEF for 10 ppb increases in NO_2_/Ox were estimated using 3-pollutant model adjusted for age, sex, height, day of the week, temporal trends, and temperature. A: Association between PEF measured in the morning at 7 a.m. and hourly concentration of NO_2_. B: Association between PEF measured in the evening at 7 p.m. and hourly concentration of NO_2_. C: Association between PEF measured in the morning at 7 a.m. and hourly concentration of Ox. D: Association between PEF measured in the evening at 7 p.m. and hourly concentration of Ox

We also show the association between hourly concentrations of other pollutants such as NO_2 _and Ox and PEF using the single-pollutant model in Figure 4. Increasing hourly concentrations of NO_2 _were positively associated with declines in both morning and evening PEF, while increasing hourly concentrations of Ox were preventively associated with declines in both morning and evening PEF. When we used the 3-pollutant model, the association between Ox and PEF disappeared. In contrast, the association between NO_2 _and PEF partially remained, even after adjustment for PM_2.5 _and Ox (Figure 5).

## Discussion

In this study in hospitalized children with severe asthma, we found a significant association between some lags of PM_2.5 _and PEF using hourly concentrations of PM_2.5 _in a single pollutant model. Some of these significant associations remained after adjustment for other air pollutants. We also found an association between 24-hour mean concentration of PM_2.5 _and PEF.

One strength of this study was that PEF was measured using an electronic spirometer under the guidance of trained nurses. PEF measurements might therefore have been more accurate than in previous studies, in most of which PEF was self-measured using a peak flow meter. In addition, our subjects were under long-term hospitalization and therefore likely maintained a more regular schedule than non-hospitalized subjects, which likely minimized the effects of unknown or unmeasurable confounders.

In this study, we found an association between PM_2.5 _and PEF in both the morning and evening using a single pollutant and a 2-pollutant model. However, this association was not stable using a 3-pollutant model. Because the effect size of the results were closely similar, the reason for the loss of a significant association was likely the strong correlation between NO_2 _and PM_2.5 _in the evening and NO_2 _and Ox in the morning (Table 3).

Similarly, we also found an association between 24-hour PM_2.5 _and PEF in both the morning and evening using the single pollutant model. We speculate that the weakened association between 24-hour PM_2.5 _and PEF using the 2- and 3-pollutant models might have also been affected by the high correlation among air pollutants.

In this study the effect size of the results for morning and evening PEF were closely similar. Several previous studies have examined the association between PM and morning PEF/evening PEF. A previous panel study in children with chronic respiratory symptoms from March through April in Finland showed that the changes in morning and evening PEF for the inter-quartile range (14 μg/m^3^) of PM_2.5 _on the previous day were -1.06 l/minute (p < 0.05) and -0.43 l/minute (not statistically significance [N.S.]), respectively [7]. With respect to other PM, such as suspended PM (SPM), which is the concentration of PM with a 100% cut-off aerodynamic diameter of ≤10 μm; or PM_10_, which is the concentration of PM with a 50% cut-off aerodynamic diameter of ≤10 μm, their association with PEF has also been shown to be strong in the morning. A panel study of children with chronic respiratory symptoms in the Netherlands showed that the changes in morning and evening PEF for 10-μg/m^3 ^increases in PM_10 _were -0.41 l/minute (p < 0.05) and -0.28 l/minute (N.S.), respectively, in children in winter [8]. A panel study of Mexican children with mild asthma showed that changes in morning and evening PEF for 20-μg/m^3 ^differences in PM_10 _were -1.37 l/minute (N.S.) and -0.53 l/minute (N.S.), respectively, in April through July and November through February [9]. A panel study of children with asthmatic symptoms in Finland showed that the changes in morning and evening PEF for the inter-quartile range (13 μg/m^3^) of PM_10 _on the same day were -0.73 l/minute (N.S.) and -0.09 l/minute (N.S.), respectively, from February through April [10]. A panel study of children with chronic respiratory symptoms in Finland showed that the changes in morning and evening PEF for the inter-quartile range (31 μg/m^3^) of PM_10 _on the previous day were -1.01 l/minute (N.S.) and -0.33 l/minute (N.S.), respectively, in March through April [7]. On the other hand, A large-scale panel study in European countries examined daily concentrations of PM_10 _and PEF in children with chronic respiratory symptoms in winter, and showed changes in morning and evening PEF for 10-μg/m^3 ^increases in PM_10 _of 0.01 l/minute (N.S.) and -0.06 l/minute (p < 0.05), respectively [11]. The authors speculated that results patterns on morning/evening PEF changes which were associated with air pollution were not clear. In most of these previous studies, the associations were evaluated using a single pollutant model.

A Japanese panel study showed that the change in morning PEF for a 10-μg/m^3 ^increase in the 3-hour concentration of SPM measured at 2 a.m. to 5 a.m. on the same day was -0.76 l/minute (p < 0.05) among children with asthma in April through September using a single pollutant model [4]. These results were robust after adjustment for NO_2 _and ozone. This study also found that declines in evening PEF were weakly associated with increasing 3-hour concentrations of SPM, albeit without statistical significance. On the other hand, when the association was examined in October through March, an increase in SPM was not associated with a decline in PEF. We speculate that the reason for this is the high correlation among air pollutants in the winter.

It seems that both morning and evening PEF were associated with concentrations of PM_2.5 _from 5 p.m. to 5 a.m. (Figure 2A and Figure 3A). We speculate that one reason for this is that exposure measurement during the night would be more representative of exposure to air pollutants, because this time point might be less influenced by the children's activity or the chance of local sources of pollution such as automobile traffic outside the window where the monitor placed. Another reason might be differences in correlations among air pollutants at night from those in the day (Table 3).

With respect to other pollutants, increasing hourly concentrations of NO_2 _were positively associated with declines in PEF using the single-pollutant model, and increasing hourly concentrations of Ox were preventively associated with declines in PEF. Use of the 3-pollutant model resulted in the loss of the association between Ox and PEF. On the other hand, the association between NO_2 _and PEF remained, even after adjusted for PM_2.5 _and Ox. One explanation of these results is the negative correlation between NO_2 _and Ox (Table 3). Because high correlations were also seen between NO_2 _and PM_2.5 _(r = 0.54 - 0.76) (Table 3), results from multi-pollutant model were likely affected by multicollinearity, and it was difficult to clarify the independent effects of NO_2 _and PM_2.5 _on PEF. Both might have come from the same sources and been subject to the same meteorological conditions. A comprehensive understanding of the complex association between air pollutants and PEF awaits further study.

### Limitations

The results of this study should be viewed cautiously for several reasons. First, the small sample size and restriction of patients to those with severe asthma may have produced problems with external validity in the selection of subjects. The subjects of this study were hospitalized children with poorly controlled asthma and frequent exacerbations. Generalization of these results to other populations, such as children with mild asthma or hospitalized children with other diseases is likely difficult. Second, ambient concentrations of air pollution might have acted as surrogate measures of exposure to other agents or specific pollution sources that were, in fact, responsible for the observed association between PM_2.5 _and PEF. Third, we assessed the exposure level of air pollutants using data from the nearest fixed monitoring station from the hospital, and not individual exposure. Exposure level might therefore be biased. However, many studies in this field use air pollutant data from fixed monitoring stations. Subjects of this study were hospitalized children. During hospitalization, they had the opportunity to expose themselves to outdoor air. They went to school twice a day, morning and afternoon. The school was next to the hospital, requiring them to walk outdoors, and they also played outdoors after school. The hospital was not equipped with a central air conditioning system at the time the study was conducted, and hospital rooms were usually ventilated by opening windows. However, these individual exposure-related conditions were not quantified. Fourth, the model assumed that the effects of PM_2.5 _would be linear over the observed range of exposure.

In this study, we tested the association between air pollutants and PEF using multiple time lags in both single and multiple pollutant models, raising the possibility of issues with repeated significance testing. We did not attempt to counter this potential problem, however, considering instead that the elevated risks of air pollutants in this study should be demonstrated to the greatest extent possible in accordance with the precautionary principle.

### Policy implications

The subjects were treated in hospital with medications which included inhaled corticosteroids. Even in this situation, we found large changes in PEF with increased PM_2.5_. Efforts to prevent the exacerbation of asthma due to air pollution should focus on air-quality standards for particulate matter based on not only on 24-hour mean concentrations but also hourly data.

## Conclusion

Among hospitalized children with severe asthma, increased hourly concentrations of PM_2.5 _were associated with a decrease in PEF.

## Abbreviations

CI: confidence interval; GEE: Generalized Estimating Equations; NO_2_: nitrogen dioxide; Ox: photochemical oxidants; PEF: peak expiratory flow; PM: particulate matter; PM_10_: particulate matter with a 50% cut-off aerodynamic diameter of ≤10 μm; PM_2.5_: particulate matter with a 50% cut-off aerodynamic diameter of ≤2.5 μm; SPM: suspended particulate matter.

## Competing interests

The authors declare that they have no competing interests.

## Authors' contributions

MS and TN designed and initiated this study. MS, MA, HW and TN were responsible for collecting the clinical data. SY and HN were responsible for creating database and statistical analysis. SY and MS were responsible for writing the draft version of manuscript. All authors commented on approved the final manuscript.
